# Effect of Pimobendan on NT-proBNP and c troponin I before and after a submaximal exercise test in dogs with preclinical mitral valve disease without cardiomegaly – a randomised, double-blinded trial

**DOI:** 10.1186/s12917-019-1980-z

**Published:** 2019-07-09

**Authors:** Nayeli Iwanuk, Ingo Nolte, Leona Wall, Maximiliane Sehn, Jonathan Raue, Anna Pilgram, Katja Rumstedt, Jan-Peter Bach

**Affiliations:** Small Animal Clinic, University of Veterinary Medicine Hanover, Foundation, Bünteweg 9, D-30559 Hannover, Germany

**Keywords:** Dog, Pimobendan, Cardiac biomarkers, Mitral valve disease, Exercise test

## Abstract

**Background:**

Exercise testing in conjunction with measurement of cardiac biomarkers NT-proBNP and cTnI is a useful tool for monitoring the effect of treatment on cardiac patients. Administering Pimobendan in dogs with degenerative mitral valve disease (DMVD) and cardiomegaly results in delaying the onset of clinical symptoms and prolonging life. Its effect in dogs with DMVD without cardiomegaly has not been well examined. The aim of the current study was to investigate the effect of administering Pimobendan in dogs with DMVD without cardiomegaly using exercise testing in conjunction with measuring cardiac biomarkers in addition to echocardiography.

Twenty-one dogs with asymptomatic DMVD without echocardiographic signs of cardiomegaly participated in a randomised, double-blinded trial. Dogs were divided into a Pimobendan-group (*n* = 11) and a placebo-group (*n* = 10) in a double-blinded study design and underwent a standardised submaximal exercise test (SSET). One dog in the Pimobendan-group was retrospectively removed from the study after being diagnosed with Leishmaniosis. Cardiac biomarkers NT-proBNP and cTnI were measured before and after exercise. Follow-up appointments were performed at days 90 and 180.

**Results:**

Dogs in the Pimobendan-group had significantly lower post-exercise NT-proBNP-levels after being administered Pimobendan than at the beginning of the study. They also had lower pre- and post-exercise-NT-proBNP-levels than those dogs in the placebo-group. There was neither a significant difference regarding the measured cTnI levels nor an increase in cTnI between the groups at any time.

**Conclusions:**

Pimobendan lowers NT-proBNP in dogs with presymptomatic mitral valve disease without cardiomegaly before and after submaximal exercise. This indicates a reduction in cardiac wall stress. If dogs with asymptomatic DMVD without cardiomegaly benefit from treatment with Pimobendan (for example, through a longer survival time) warrants further investigation.

## Background

Biomarkers are measurable indicators which can be used to examine an organ function, a pathological process or responses to therapeutic intervention [1]. An optimal biomarker is easy to measure (preferably non-invasively) and should have a high specificity for one organ [2]. Cardiac biomarkers, N-terminal pro natriuretic peptide type B (NT-proBNP) and cardiac troponin-I (cTnI), are gaining in importance as diagnostic and prognostic tools in cardiac diseases in dogs [3–5].

Brain natriuretic peptide (BNP) is a hormone, which is released into the blood circulation due to increased myocardial wall stress [3, 6]. Its precursor, the pro-hormone (pro-BNP), is produced by myocardiocytes and its production is increased in chronic heart disease patients [3, 7]. Upon release into the blood, pro-BNP is cleaved into the active BNP and the inactive NT-proBNP [7]. BNP causes vasodilatation, inhibition of sympathetic nervous system, inhibition of renin-angiotensin-aldosterone-system (RAAS), natriuresis and diuresis [7]. Thus, BNP lowers the pre- and afterload. Due to its longer half-life, NT-proBNP is better suited as a diagnostic parameter [7]. NT-proBNP positively correlates with the grade and prognosis of the disease in dogs with asymptomatic degenerative mitral valve disease (DMVD) and can be used as an additional diagnostic tool in dogs with heart murmur and symptoms which have cardiac or non-cardiac causes [8]. It may be influenced by non-cardiac diseases like acute or chronic kidney disease, pulmonary hypertension or hyperthyroidism [3, 8].

Cardiac troponin-I (cTnI) is a sensitive marker of myocardial damage and necrosis [3, 9]. It is expressed in cardiac muscle tissue and is a sensitive marker of damage to the heart muscle. However, it is also elevated in chronic renal failure or other systemic illnesses and increases with age [3, 9–11]. In humans and canines, it is released into the circulation four to twelve hours after acute damage [9, 12, 13]. cTnI is not increased in all individuals with mild to moderate DMVD [3, 14].

Cardiac biomarkers have been used in conjunction with exercise testing in human cardiac patients to improve risk stratification [15–17]. Magne et al. (2012) observed that asymptomatic patients with DMVD with elevated BNP-levels after exercise have an increased incidence of cardiac events. Sinha et al. (2016) observed that asymptomatic patients with elevated BNP-values after exercise have a high risk of reduced event-free survival, and concluded that measuring exercise BNP might have prognostic potential in addition to echocardiographic data. The exercise test had to be stopped in 66 cases because of dyspnoea. These patients had similar resting BNP-values compared to others but significantly higher exercise BNP-values [17].

In a recent study in which different parameters (cardiac biomarkers NT-proBNP and cTnI, heart rate, lactate, acid-base-status, respiratory frequency, blood gases, echocardiography) were measured in combination with exercise testing in dogs, cardiac biomarkers showed the most reliable results [14]. Therefore, exercise in a standardised submaximal treadmill test (SSET) induces different changes in cardiac biomarkers in healthy dogs and in those animals with asymptomatic DMVD. The increase after exercise is higher in dogs with DMVD than in healthy dogs. The combination of measuring cardiac biomarkers and exercise testing could thus become a helpful diagnostic tool [14]. However, it is still unclear if the combination of an exercise test and measuring cardiac biomarkers is suitable to monitor therapeutic effects of cardiac medication and to adjust the treatment.

A positive effect of treatment with Pimobendan for symptomatic and asymptomatic DMVD patients with cardiomegaly is well established. Nevertheless, there is still a lot of uncertainty regarding the treatment of dogs with asymptomatic DMVD without signs of cardiomegaly (CHIEF B1) [18–22].

The aim of the current study was to evaluate the combination of exercise testing and cardiac biomarkers as a tool for monitoring the effect of cardiac treatment in dogs. The effect of Pimobendan on cardiac biomarkers as an indicator of the progression of early stage DMVD should be observed to obtain objective information regarding the therapy with Pimobendan in these patients.

## Materials and methods

### Trial design

This study was a prospective, double-blinded, randomised placebo-controlled trial. Originally, eleven dogs were administered with Pimobendan and ten dogs were administered with placebo. One dog in the Pimobendan-group completed the study, but was retrospectively removed from the study due to the diagnosis of Leishmaniosis, which is known to affect cardiac biomarker measurements [23]. The study was approved by an ethical review committee (Lower Saxony State Office for Consumer Protection and Food Safety, 33.9–42,502-05-14A484) and written consent from the dog owners was obtained. This study adheres to the CONSORT Guidelines.

### Dogs

Only dogs with asymptomatic DMVD without signs of cardiomegaly or congestive heart failure were eligible for participation in the study. Dogs were required to have a typical heart murmur, valvular lesions on the mitral valve and mitral regurgitation depictable by echocardiographic examination (Vivid E9, GE Healthcare, Solingen, Germany). Dogs had to be free of signs of congestive heart failure and cardiomegaly had to be excluded by assessing the LA/Ao ratio (Ratio of diameters of left atrium and aortic root ≤1.6) and LVIDDN (diastolic diameter of left ventricle ≤1.7) by echocardiography. LA/Ao ratio was measured as described by Hansson et al. [24]. Normalised dimension of LVIDDN was calculated according to the following formula: LVIDDN = LVIDd (cm)/(BW (kg))^0.294^ [25]. Dogs with raised LA/Ao ratio and raised LVIDDN were excluded from the study. If only one parameter was above the reference range, dogs were eligible to participate in the study. Dogs with other severe systemic diseases or orthopaedic symptoms and those animals unwilling to run on the treadmill (“quasar”, h/p/cosmos sports and medical GmbH, Nussdorf-Traunstein, Germany) were excluded from the study.

For every dog included in the study, the patient’s history was taken and a thorough clinical examination was performed. In addition to this, blood samples were collected and a complete blood count and clinical chemistry were assessed to exclude non-cardiac diseases. Dogs were classified in accordance with the modified CHIEF system [22] after echocardiographic examination. Before running on the motorised treadmill, a ten-minute electrocardiogram was performed to detect possible arrhythmia. Before and after running on the treadmill, blood was sampled to measure the cardiac biomarkers cTnI and NT-proBNP, lactate (Cobas c 311 Analyser Roche Diagnostics, Mannheim, Germany), electrolytes (Rapidlab 1260, Siemens Healthcare Diagnostics GmbH, Eschborn, Germany) and acid-base state (Rapidlab 1260, Siemens Healthcare Diagnostics GmbH, Eschborn, Germany).

These examinations were performed on admittance to the clinic and at every follow-up appointment at days 90 and 180. At day 180, the primary end-point was reached. If dogs showed any signs of intolerance regarding Pimobendan or placebo, the secondary end-point was reached. During the entire study-period and after the study, the dogs lived with their owners.

Data were collected at the Small Animal Clinic, University of Veterinary Medicine Hannover, Foundation, Germany. Sample size was determined by a power analysis.

### Blinding, randomisation and allocation

Dogs were divided into group A (Pimobendan-group) and group B (placebo-group) by drawing lots. A ten-block randomisation was used to assign dogs to either of the groups. The last dog was randomised by means of two-block randomisation. Individuals who had no other role in the study generated the random allocation sequence, assigned participants and kept the blinding code until data analysis had been completed. The investigators, owners and statistician were blinded to the treatment allocation.

### Trial medication

The trial medication Pimobendan was administered by the owner at a mean (± standard deviation (SD)) dose of 0.41 (± 0.05) mg/kg per day. The medication was administered every 12 h, always 1 hour before feeding. The placebo contained lactose and looked similar to the Pimobendan capsules. No other cardiac treatment was allowed for the duration of the study.

### Standardised, submaximal exercise test (SSET)

**Fig. 1:**
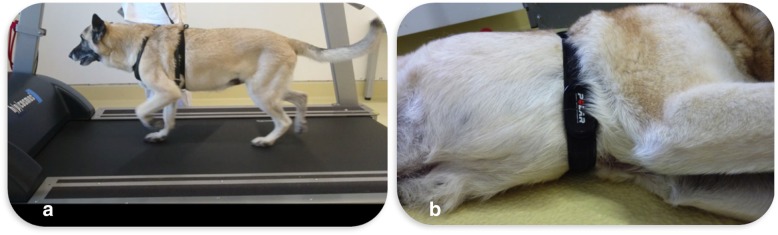
SSET and correct position of the electrode belt **a**) Dog running on the treadmill with an incline of 0% and a speed of 6.4 km/h (stage 1). The electrode belt for telemetric measurement of heart rate is visible behind the chest belt. **b**) Correct position of the Polar® heart rate monitor. The electrodes are on the left and on the right side of the thorax and the transmitter is located ventrally

An SSET was used [14]. For the SSET, dogs had to run on a motorised treadmill (“quasar”, h/p/cosmos sports and medical GmbH, Nussdorf-Traunstein, Germany). Before the test started, there was a short familiarisation period. The dogs were motivated by food or a toy to trot on the treadmill. All dogs ran at their own comfort speed (mean 5.5 ± 1.3 km/h). The SSET included six stages. Every stage lasted 3 minutes. The initial incline on the treadmill was 0%. The incline was increased for every subsequent stage in steps of 4%, reaching 20% by stage 6 in the course of the exercise test. After stages 1, 3 and 5 had been completed, there was a 20-s break and after stages 2 and 4 there was a 3-min break. The heart rate was measured continuously by a Polar® heart rate monitor (Fig. 1; Polar FT7N and Polar H1, Polar Electro GmbH Deutschland, Büttelborn, Germany). Before and after running, the respiratory frequency and the rectal body temperature were measured. These measurements were repeated 5 minutes after finishing the SSET. If the dog showed any signs of overstraining or if the heart rate was 240 bpm or higher, the SSET was immediately stopped. To ensure a minimal workload, the heart rate had to be raised by 40% compared to the heart rate at rest. At every follow-up appointment, the dogs ran at the same speed as at the first appointment.

### Cardiac biomarkers

NT-proBNP was measured from 0.3 mL EDTA-Plasma and cTnI from 0.5 mL serum samples. The samples were stored in a freezer at − 80 °C until being sent to IDEXX-laboratories (Ludwigsburg, Germany). The Cardiopet® proBNP test was used to measure NT-proBNP and the high-sensitive Troponin I test to measure cTnI.

### Statistical methods

The Shapiro-Wilk test was used to test for standard distribution of the data. Since the data showed no standard distribution, the Mann-Whitney test was chosen to compare the biomarker values of both groups on the respective days before and after exercise and the Wilcoxon’s signed rank test was chosen to compare the increase after exercise of each group on the respective days and to compare the values within the groups. The minimal level of significance was set at < 0.05. Spearman’s correlation coefficients were calculated to test for correlation between the echocardiographic parameters LA/Ao and LVIDDN and the measured cardiac biomarkers before and after exercise.

## Results

### Participant flow

**Fig. 2:**
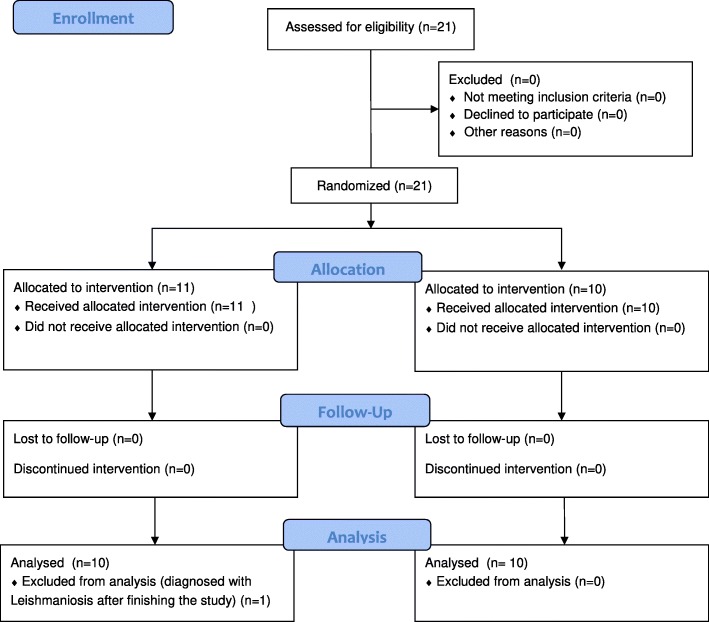
A flow chart indicating the outcome of 21 dogs randomised in the current study. The number of participants which were randomly assigned, received intended treatment and were analysed for primary outcome

A flow chart indicating the outcome of 21 dogs randomised in the current study is shown in Fig. 2.

### Baseline characteristics

The baseline characteristics of the included dogs are shown in Table 1 and an overview of different variables in the Pimobendan-group and placebo-group is shown in Table 2.

**Table 1:** Characteristics of the included dogs

Number	Breed	Gender	Age (years)	Weight (kg)	Heart murmur	Speed (km/h)	Group
1	mixed breed	mn	11.1	31.6	II/VI	5.5	A
2	mixed breed	mn	10.8	16.6	I/VI	5.5	A
3	mixed breed	mn	9.8	16.0	I/VI	6.3	A
4	mixed breed	fn	10.8	26.6	I/VI	6.4	B
5	mixed breed	fn	15.7	8.7	IV/VI	4.0	B
6	mixed breed	fn	7.7	11.9	II/VI	4.5	B
7	mixed breed	fn	6.2	6.8	I/VI	4.4	A
8	Golden Retriever	fn	8.5	34.5	I/VI	7.0	B
9	Golden Retriever	mi	8.1	28.7	II/VI	6.5	B
10	Jack Russel Terrier	mn	13.5	12.0	IV/VI	3.0	A
11	Jack Russel Terrier	mn	12.4	7.6	II/VI	4.1	A
12	Beagle	fn	7.9	18.8	II/VI	7.0	B
13	Beagle	mn	6.2	18.8	IV/VI	6.3	A
14	Cavalier King Charles Spaniel	fn	6.8	8.0	IV/VI	5.5	A
15	Entlebuch cattle dog	fn	5.8	18.0	I/VI	6.0	B
16	Small Munsterlander	fn	11.8	19.8	I/VI	6.7	A
17	Pekinese	mn	6.2	8.9	I/VI	3.4	A
18	West Highland White Terrier	fn	7	6.9	I/VI	3.0	B
19	Poodle	mn	12.2	10.6	II/VI	4.5	B
20	American Staffordshire Terrier	mn	7.8	16.5	I/VI	5.3	B

Breed, gender, age, weight, heart-murmur, speed running on the treadmill and group (A/B) of each included dog

mn: male neutered; fn: female neutered; mi: male intact. There were ten female dogs (all neutered) and ten male dogs (one intact, nine neutered). The mean age was 8.3 (± 2.8) years and the mean weight was 16.3 (± 8.4) kg. Regarding the intensity of the heart murmur, participants were distributed as follows: grade 1 (*n* = 10), grade 2 (*n* = 6), grade 3 (*n* = 0) or grade 4 (*n* = 4) of six possible grades. Dogs in group A were administered Pimobendan; dogs in group B were administered the placebo

**Table 2:** Overview of different variables in Pimobendan- and placebo-group

Variable	Pimobendan	Placebo
Number	10	10
Gender (M/F)	7/3	3/7
Age (years, mean ± SD)	10.3 ± 2.8	8 ± 2.8
Weight (kg, mean ± SD)	14 ± 7.7	17.3 ± 8.7
Murmur (I/II/III/IV)	5/2/0/3	5/4/0/1
Speed (km/h, mean ± SD)	5.5 ± 1.3	5.7 ± 1.3

This table shows the number of dogs (n), gender (M/F), age (years; mean ± standard deviation (SD)), weight (kg; mean ± SD), heart murmur (grades I-VI/VI), and the speed running on the treadmill (km/h; mean ± SD) of Pimobendan- and placebo-group. The Pimobendan dose (mg/kg/d; mean ± SD) is also shown

Dogs were recruited from November 2016 and the last follow-up appointment took place in March 2018.

### Echocardiography

No dog showed an increased LA/Ao-ratio at the initial examination, but one dog in the control-group developed an increased LA/Ao-ratio at day 180. Two dogs in the Pimobendan-group and two dogs in the control-group showed slightly increased LVIDDN-values at the initial examination. Two of these dogs still had LVIDDN above the reference range at the final examination, two dogs’ LVIDDN decreased to be within the reference range. One of these dogs was in the Pimobendan-group and the other dog in the placebo-group.

There was no statistically significant difference regarding the LA/Ao-ratio and LVIDDN between the groups or between different examinations. Values for the LA/Ao-Ratio and LIVDDN for all dogs at all time-points of the examination can be found in Table 3.

**Table 3:** LA/Ao-Ratio and LVIDDN of all dogs at the different examination times

Day 0		Day 90		Day 180		
LA/Ao	LVIDDN	LA/Ao	LVIDDN	LA/Ao	LVIDDN	Group
1.22	1.42	1.22	1.57	1.35	1.36	A
1.00	1.73	1.37	1.55	1.39	1.45	A
1.22	1.46	1.33	1.33	1.33	1.37	A
1.15	1.43	1.15	1.26	1.15	1.26	A
0.95	1.41	1.30	0.97	1.18	1.04	A
1.43	1.61	1.22	1.52	1.20	1.42	A
1.11	1.78	1.27	1.81	1.06	2.00	A
1.27	1.60	1.23	1.41	1.53	1.58	A
1.46	1.51	1.32	1.52	1.34	1.59	A
1.35	1.70	1.46	1.63	1.31	1.75	A
1.07	1.65	1.50	1.65	1.51	1.70	B
1.24	1.62	1.11	1.40	1.09	1.63	B
1.30	1.55	1.30	1.67	1.22	1.42	B
1.51	1.76	1.40	1.55	1.70	1.60	B
1.17	1.67	1.47	1.73	1.30	1.72	B
1,28	1.58	1.34	1.40	1.49	1.50	B
1,22	1.56	1.06	1.66	1.40	1.38	B
1,29	1.86	1.13	1.60	1.38	1.79	B
1,30	1.63	1.41	1.42	1.38	1.67	B
1,42	1.34	1.42	1.28	1.25	1.26	B

### Cardiac biomarkers

The mean change of NT-proBNP (pmol/L) after exercise at days 0, 90 and 180 in the Pimobendan-group and placebo-group is shown in Table 4.

**Table 4:** Mean (± SD) change of NT-proBNP (pmol/l) after exercise on days 0, 90 and 180

Change after exercise	Day 0	Day 90	Day 180
Pimobendan-group	81.5 (± 89.9)	56.0 (± 75)	−10 (± 39.09)
Placebo-group	120.7 (± 109)	137.7 (± 164.9)	152.2 (± 159.4)

**Fig. 3:**
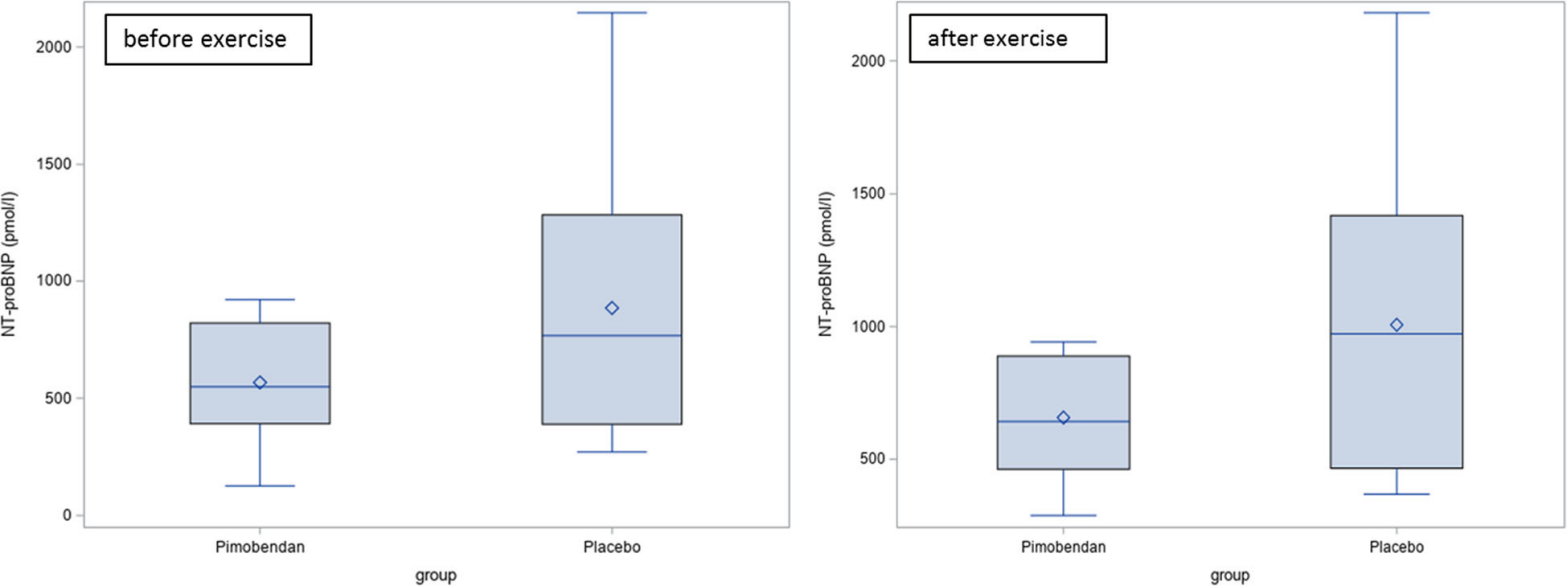
NT-proBNP-value at day 0 before and after exercise. There is no significant difference between the Pimobendan-group and the placebo-group (before exercise: *p* = 0.15, after exercise: *p* = 0.10)

At day 0 there was no significant difference between the Pimobendan-group and the placebo-group before and after exercise regarding the NT-proBNP-value (Fig. 3; before exercise: *p* = 0.15, after exercise: *p* = 0.10).

**Fig. 4:**
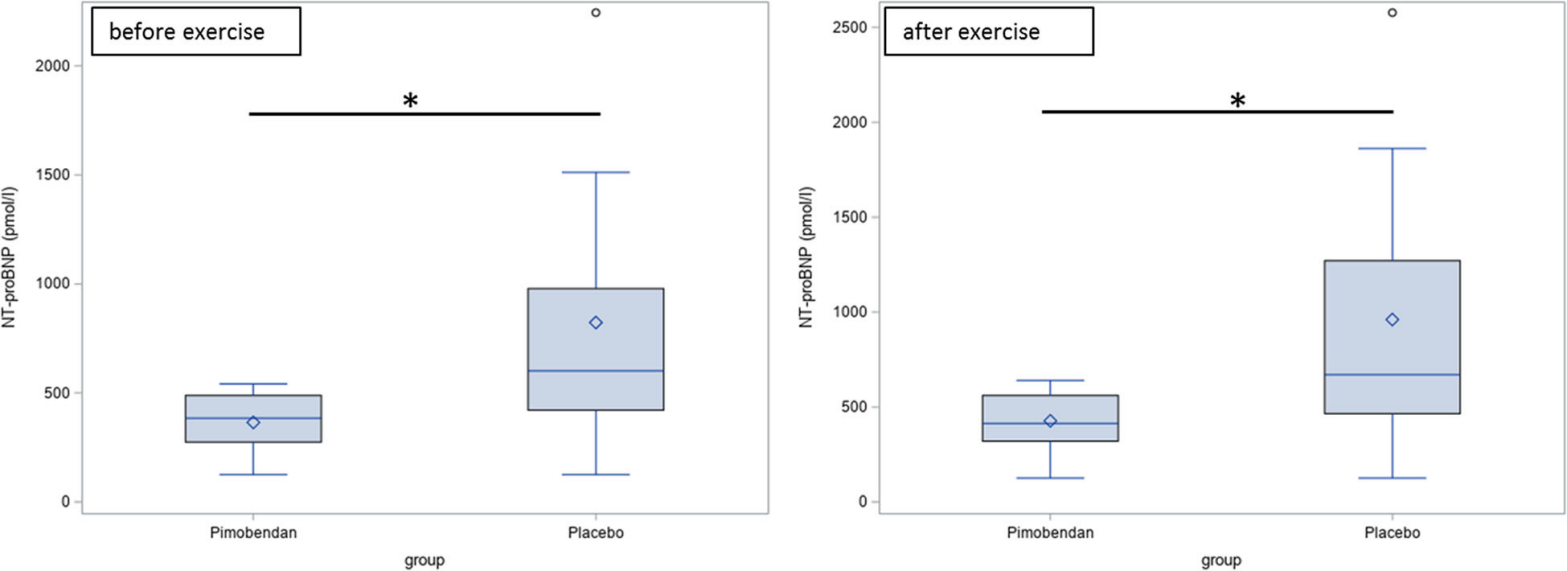
NT-proBNP-value after 90 days before and after exercise. There is a significant difference (*) between the Pimobendan-group and the placebo-group (before exercise: *p* = 0.031, after exercise: *p* = 0.034)

At day 90, the Pimobendan-group showed a significantly lower NT-proBNP-value after exercise than at day 0 (*p* = 0.031). At day 90, the Pimobendan-group had a significantly lower NT-proBNP-value before and after exercise regarding the NT-proBNP-value than the placebo-group (Fig. 4; before exercise *p* = 0.031, after exercise *p* = 0.034).

**Fig. 5:**
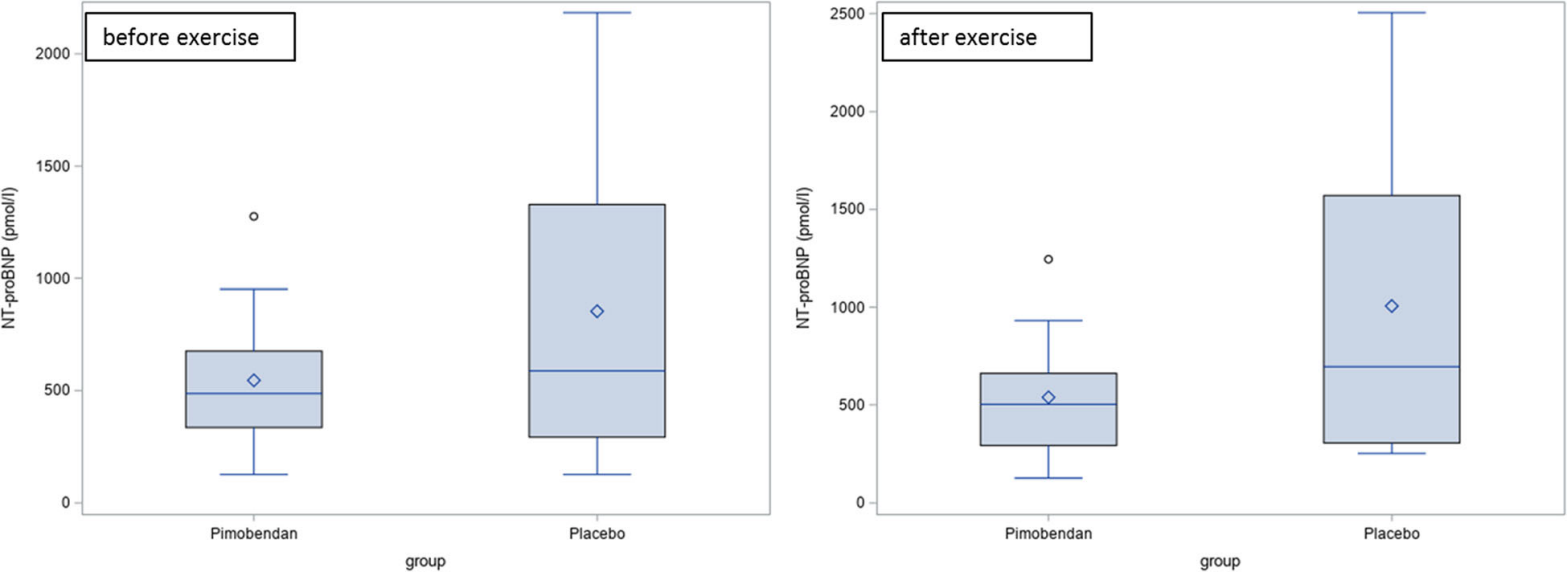
NT-proBNP-value at day 180 before and after exercise. There is no significant difference between the Pimobendan-group and the placebo-group (before exercise: *p* = 0.59, after exercise: *p* = 0.34)

At day 180, there was no significant difference between the Pimobendan-group and the placebo-group before and after exercise (Fig. 5; before exercise: *p* = 0.59, after exercise: *p* = 0.34).

**Fig. 6:**
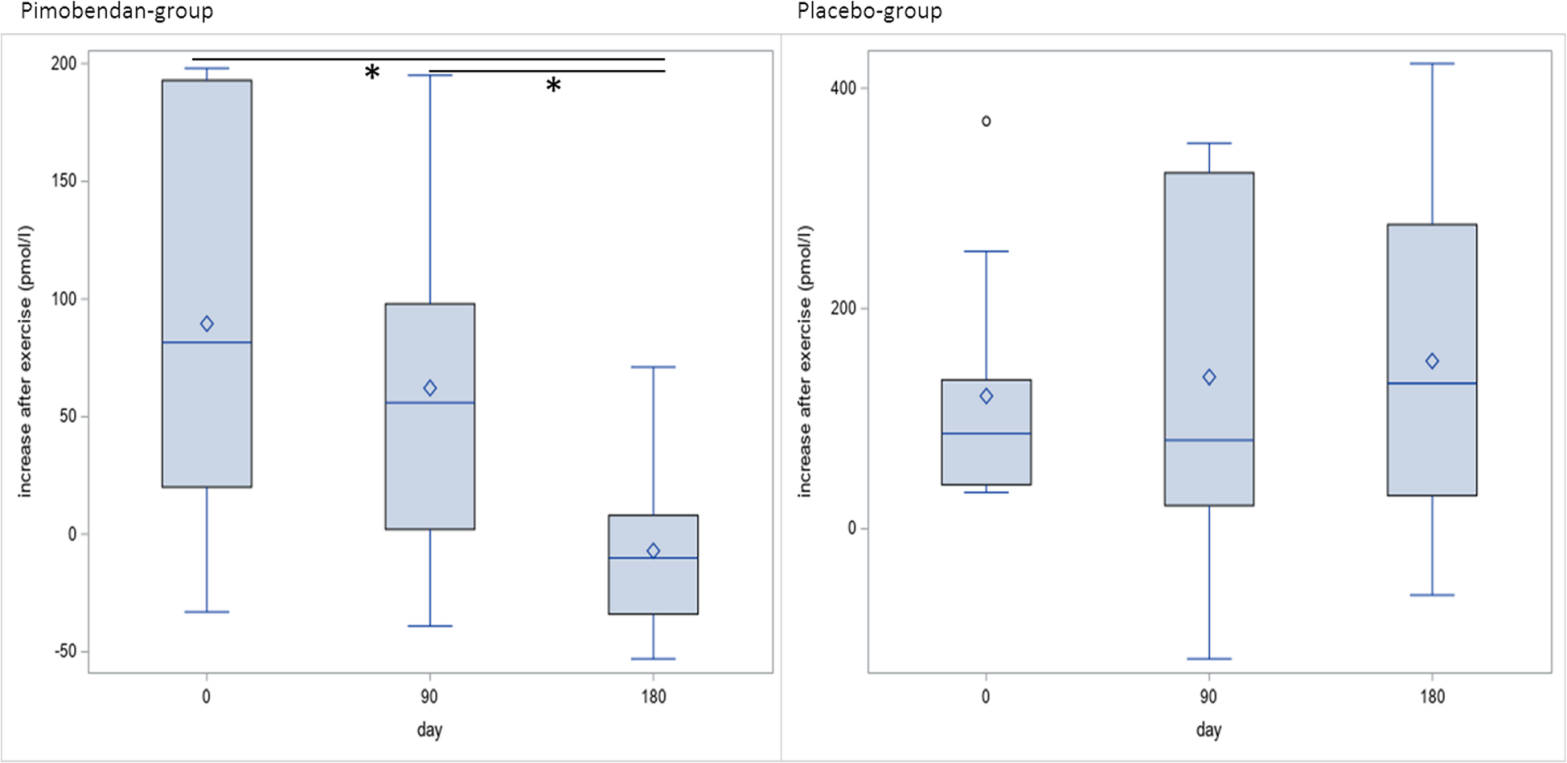
Changes after exercise in NT-proBNP in the Pimobendan- and placebo-group. There is a significant difference (*) in the Pimobendan-group between day 0 and day 180 (*p* = 0.014) and between day 90 and day 180 (*p* = 0.047). There is no significant difference in the placebo-group

The change after exercise of NT-proBNP in the Pimobendan-group was significantly lower at day 180 than at days 0 and 90 (Fig. 6; days 0 and 180 *p* = 0.014, days 90 and 180 *p* = 0.047).

**Fig. 7:**
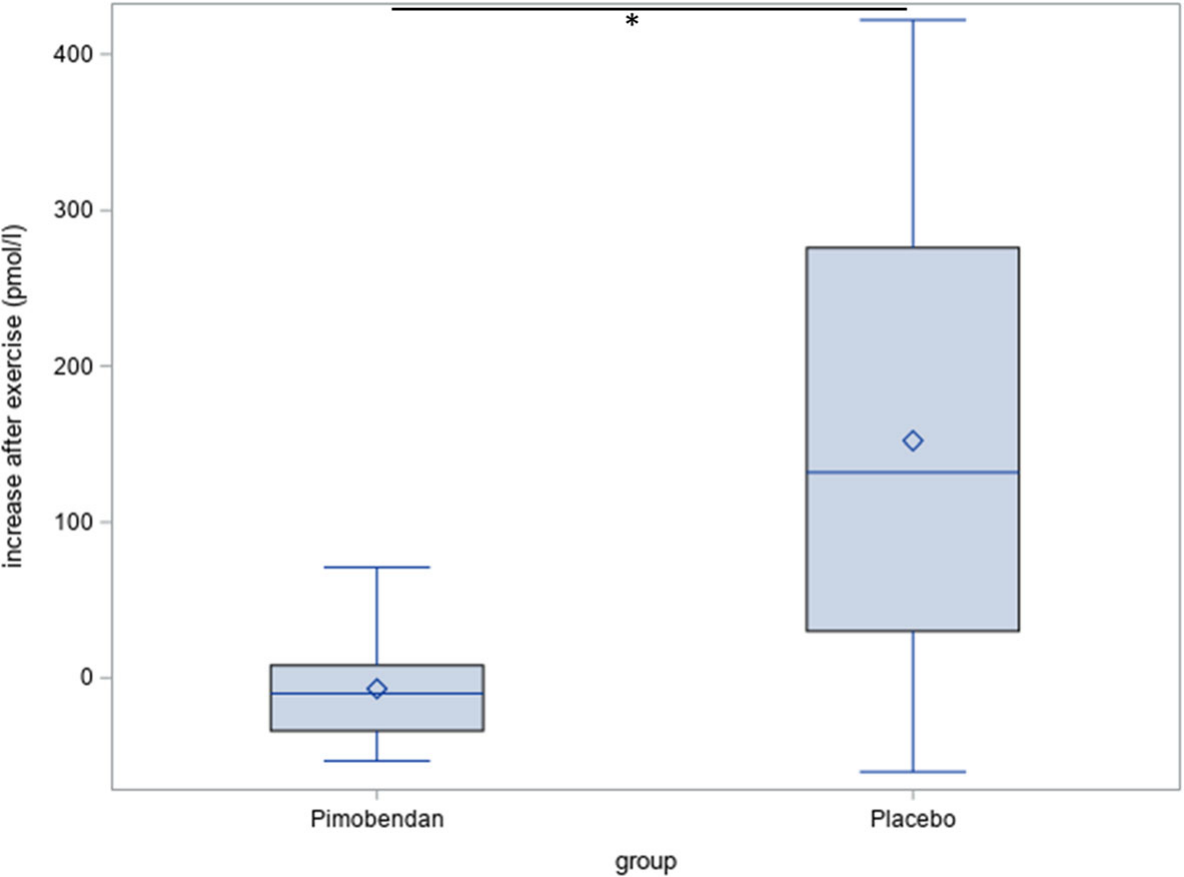
Changes after exercise in NT-proBNP. There is a significant difference (*) between the Pimobendan-group and the placebo-group at day 180 (*p* = 0.03)

The change after exercise of NT-proBNP-values at day 180 was significantly lower in the Pimobendan-group than in the Placebo-group (Fig. 7; *p* = 0.03).

The mean (± SD) cTnI-value (ng/mL) in the Pimobendan-group and placebo-group before and after exercise is shown in Table 5.

**Table 5:** Mean (± SD) cTnI-value (ng/mL) in the Pimobendan- and placebo-group before and after exercise

cTnI	Pimobendan-group		Placebo-group	
	before exercise	after exercise	before exercise	after exercise
day 0	0.022 (± 0.037)	0.051 (± 0.041)	0.1 (± 0.014)	0.116 (± 0.015)
day 90	0.039 (± 0.032)	0.048 (± 0.038)	0.049 (± 0.037)	0.057 (± 0.041)
day 180	0.038 (± 0.028)	0.041 (± 0.036)	0.051 (± 0.048)	0.068 (± 0.065)

There was neither a significant difference regarding the measured cTnI levels nor the increases in cTnI between the groups. There were also no significant differences between the different examination times within the two groups.

Spearman’s correlation coefficients for the comparison of NT-proBNP and cTnI levels before and after exercise with LA/Ao ratio and LVIDDN can be found in Table 6. There was no significant correlation between the cardiac biomarker levels and echocardiographic parameters.

**Table 6:** Spearman’s correlation coefficients for comparing cardiac biomarker levels with echocardiographic parameters

	NT-proBNP		cTnI	
	before exercise	after exercise	before exercise	after exercise
LA/Ao	−0.05	−0.05	−0.01	−0.01
LVIDDN	0.17	0.18	0.15	0.19

### Standardised submaximal exercise test

**Fig. 8:**
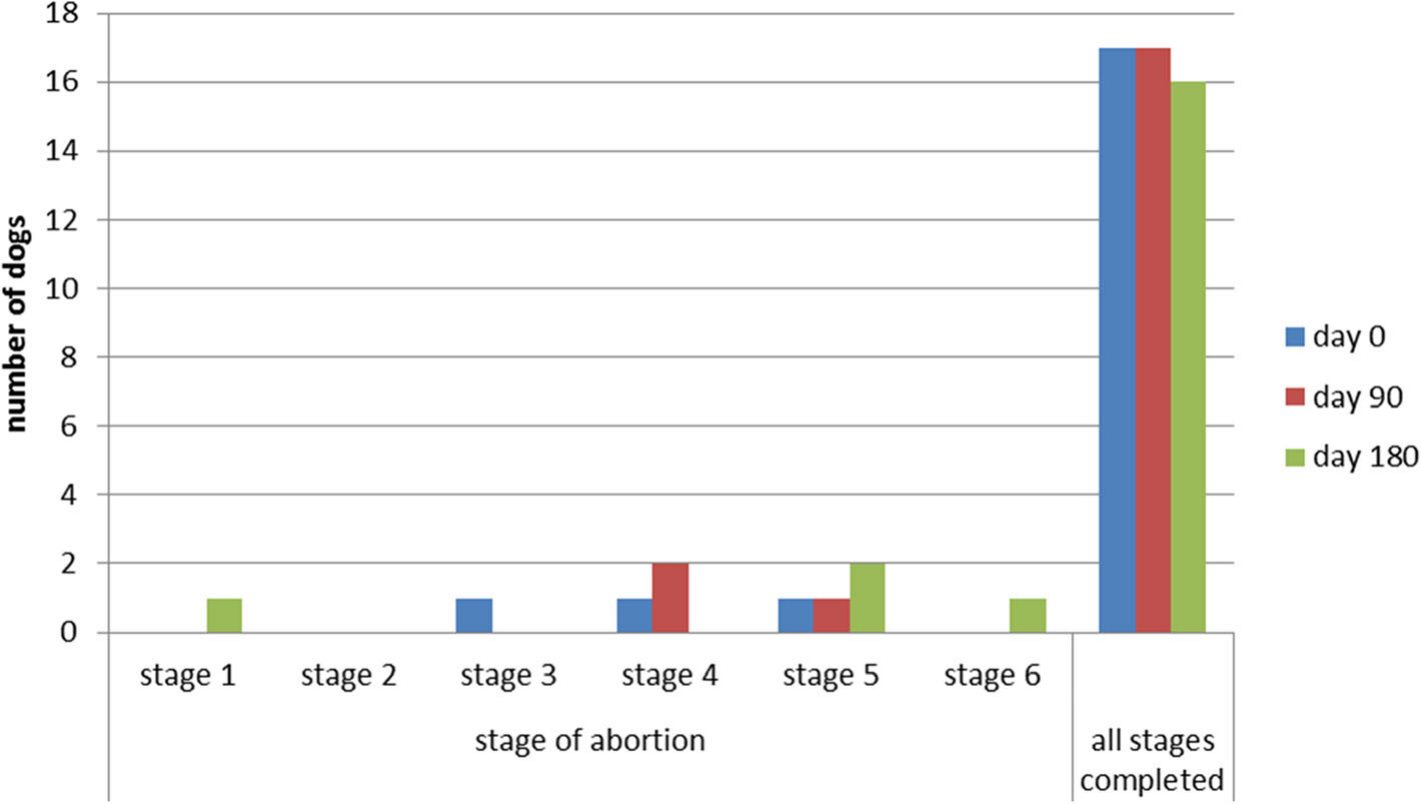
Distribution of the number of dogs aborting or completing all stages of the SSET. Distribution of the number of dogs aborting (day 0 *n* = 3, day 90 n = 3, day 180 *n* = 4) and the number of dogs completing all of the six stages (day 0 *n* = 18, day 90 n = 18, day 180 *n* = 17) of the SSET at days 0, 90 and 180. The reasons for abortion of the SSET were lameness (*n* = 1) or reluctance to run (*n* = 9). The heart rate of the dogs did not exceed 240 bpm at any point of the SSET and no dog showed signs of overstraining

The distribution of the number of dogs aborting or completing all stages of the SSET is shown in Fig. 8.

The dogs examined in the current study also participated in another study by the same authors (“Effect of Pimobendan on physical fitness, lactate and sonographic parameters in dogs with preclinical mitral valve disease without cardiomegaly”, Iwanuk et al., under review).

There was no significant difference regarding side effects between the groups.

## Discussion

Recently, the results of a large multicentre study (EPIC-study) showed that dogs with asymptomatic DMVD and signs of cardiomegaly benefit from treatment with Pimobendan with delayed onset of clinical symptoms and prolonged survival [18, 19]. The strong positive effect observed by these authors in dogs with asymptomatic disease and cardiomegaly (CHIEF B2) justifies the evaluation of the effect of Pimobendan in dogs with preclinical DMVD without cardiomegaly (CHIEF B1). A special focus of the current placebo-controlled, double-blinded prospective study was to investigate the effect of Pimobendan on cardiac biomarkers, as these represent the load on the heart. To investigate this, all patients examined in the present study underwent an SSET which was repeated after 90 and 180 days of treatment.

The results of the current study show that administering Pimobendan lowers NT-proBNP-values in dogs with asymptomatic DMVD without cardiomegaly. These results match the results of Atkinson et al. (2009) who investigated the effect of Pimobendan on NT-proBNP-levels in dogs with pulmonary hypertension secondary to mitral valve disease. Atkinson et al. (2009) also observed a reduction in NT-proBNP-levels under Pimobendan treatment in conjunction with improved quality of life. A pathological wall stress in response to volume overload is reflected by a high NT-proBNP-value in dogs [3, 5, 26]. The combination of vasodilatation and inotropic effects of Pimobendan decreases the after-load and increases the cardiac performance [27–29]. In patients with asymptomatic DMVD without cardiomegaly, no haemodynamically significant changes were observed but wall stress can be present and detected by an increased NT-proBNP-value [5, 22]. The decrease in NT-proBNP in dogs treated with Pimobendan observed in the present study might indicate a lower wall stress. In addition to this, CHIEF B1 patients in the present study treated with Pimobendan showed a decrease in NT-proBNP before and after the strain on the SSET at day 90. This finding was not confirmed at day 180. The reason for this discrepancy between the two examination times is unclear, though it is known that NT-proBNP values are subject to weekly fluctuations even in healthy dogs [30]. There was no significant difference regarding the echocardiographic parameters LA/Ao and LVIDDN between the groups or the examination times. These results match the findings of Chetboul et al. (2007) who found no changes in the left ventricular diastolic diameter and LA/Ao ratio due to Pimobendan therapy [21]. In comparison to this, LA/Ao and LVIDDN significantly decreased after treatment with Pimobendan in the EPIC-study [19]. Possible reasons for this discrepancy in the results may be due to the advanced stage of the disease in the EPIC-study or to the lower animal number in the present study and the study of Chetboul et al. [21]. Regarding the correlation analysis of cardiac biomarker levels and echocardiographic parameters in the present study, no correlation was observed. This finding was expected since all dogs participating in the study were in the same early stage of DMVD.

While the positive effect of Pimobendan in dogs with symptomatic DMVD has been verified for several years now [31, 32], the use of Pimobendan in dogs with asymptomatic DMVD has been subject to discussion. There have been some critical reports regarding the use of Pimobendan in patients with asymptomatic DMVD in the past [20, 21]. Among the reported effects was the increase in mitral valve regurgitation under therapy with Pimobendan or the decrease in mitral valve regurgitation after switching therapy from Pimobendan to Benazepril [20, 21]. In addition to this, Chetboul et al. (2007) reported more severe histological lesions (irregular and thickened mitral valve, focal haemorrhages beneath the endocardium of the mitral valve leaflets) in animals treated with Pimobendan in comparison to Benazepril. Even though these reports provide interesting information regarding possible effects of Pimobendan application, there is no evidence regarding the influence of the medication on the survival time or onset of clinical symptoms.

In contrast to the study of Chetboul et al. (2007), in which myocardial damage was reported, cTnI was not influenced by Pimobendan in the present study. This indicates that there were no structural cardiac lesions after administering Pimobendan in dogs with early stage mitral valve disease. Furthermore, no increase in regurgitation was observed by the echocardiographic examinations during the 180-day treatment.

In addition to measuring pre-exercise NT-proBNP, blood sampling and NT-proBNP measurements were also performed after exercise. In a recent study (Wall et al. 2018), healthy dogs showed a lower increase in NT-proBNP after exercise testing than dogs with early stage DMVD. In the current study, dogs being treated with Pimobendan showed a lower increase in NT-proBNP than those in the placebo-group. This could be interpreted as a supportive effect of Pimobendan on a reduction in cardiac wall stress. As it is known that the NT-proBNP-value is correlated with the prognosis in dogs with asymptomatic DMVD [8], this finding of lower NT-proBNP-values might indicate a better prognosis.

Exercise also induces cardiac wall stress and an increase in NT-proBNP and plasma BNP in healthy humans. However, it remains within the physiological range [33–35]. Exercise levels of BNP (which is the active cleavage-product of pro-BNP) have prognostic potential in humans with asymptomatic DMVD [16, 17]. Sinha et al. (2016) observed that patients with cardiac events (e.g., congestive heart failure, acute pulmonary edema) had shown higher post exercise NT-proBNP-values before the occurrence of the event than those patients without subsequent cardiac events. This finding is especially important because there were no differences between the cardiac event-group and the non-cardiac event-group regarding the resting NT-proBNP-values. In the present study, there was a significant difference between the Pimobendan-group and the placebo-group regarding the change in NT-proBNP after exercise at day 180. This finding might indicate that dogs which were treated with Pimobendan and examined in the present study, have a lower risk of developing a cardiac event. The results observed in the study by Sinha et al. (2016) show that measuring NT-proBNP after exercise in addition to measuring it at rest has additional value regarding the prognosis in human cardiac patients. Thus, lower NT-proBNP-values in the Pimobendan-group observed in the current study might predict less cardiac wall stress after exercise and therefore indicate a better prognosis. To confirm this hypothesis long-term-studies combining measurement of post-exercise NT-proBNP-values and investigation of survival times and time until onset of clinical symptoms are needed.

## Limitations

One limitation of this study is the small number of dogs (*n* = 20). In addition to this, no data regarding the time to the onset of clinical symptoms or survival time were collected; both are important factors in clinical decision-making in patients with preclinical mitral valve disease.

## Conclusion

The lower NT-proBNP-value after Pimobendan treatment in dogs with presymptomatic mitral valve disease without cardiomegaly before and after submaximal exercise indicates a reduction in cardiac wall stress. The question whether dogs with asymptomatic DMVD without cardiomegaly might benefit from treatment with Pimobendan (for example, resulting in a longer survival time) warrants further investigation.

## Data Availability

All data generated or analysed during this study are included in this published article.

## Abbreviations

BNP: brain natriuretic peptide
bpm: beats per minute
CHIEF: canine heart international expert forum
cTnI: cardiac troponin I
DMVD: degenerative mitral valve disease
F: female
fn: female neutered
LA/Ao ratio: ratio of diameters of left atrium and aortic root
LVIDd: diastolic diameter of left ventricle
LVIDDN: diastolic diameter of left ventricle normalised
M: male
mi: male intact
mn: male neutered
NT-proBNP: N-terminal pro natriuretic peptide type B
pro-BNP: pro natriuretic peptide type B
RAAS: renin-angiotensin-aldosterone system
SD: standard deviation
SSET: standardised submaximal exercise test
